# Security Risk Assessment Approach for Distribution Network Cyber Physical Systems Considering Cyber Attack Vulnerabilities

**DOI:** 10.3390/e25010047

**Published:** 2022-12-27

**Authors:** Buxiang Zhou, Binjie Sun, Tianlei Zang, Yating Cai, Jiale Wu, Huan Luo

**Affiliations:** 1 College of Electrical Engineering, Sichuan University, Chengdu 610065, China; 2 Intelligent Electric Power Grid Key Laboratory of Sichuan Province, Sichuan University, Chengdu 610065, China

**Keywords:** security risk assessment, cyber physical systems, Bayesian network, common vulnerability scoring system, fuzzy analytic hierarchy process, entropy weight method

## Abstract

With the increasing digitalization and informatization of distribution network systems, distribution networks have gradually developed into distribution network cyber physical systems (CPS) which are deeply integrated with traditional power systems and cyber systems. However, at the same time, the network risk problems that the cyber systems face have also increased. Considering the possible cyber attack vulnerabilities in the distribution network CPS, a dynamic Bayesian network approach is proposed in this paper to quantitatively assess the security risk of the distribution network CPS. First, the Bayesian network model is constructed based on the structure of the distribution network and common vulnerability scoring system (CVSS). Second, a combination of the fuzzy analytic hierarchy process (FAHP) and entropy weight method is used to correct the selectivity of the attacker to strike the target when cyber attack vulnerabilities occur, and then after considering the defense resources of the system, the risk probability of the target nodes is obtained. Finally, the node loads and node risk rates are used to quantitatively assess the risk values that are applied to determine the risk level of the distribution network CPS, so that defense strategies can be given in advance to counter the adverse effects of cyber attack vulnerabilities.

## 1. Introduction

With the massive access of controllable distributed power sources and flexible loads, the integration of Internet communication technology and automatic control technology, the digitalization and informatization degree has been improving in distribution network systems, which have become typical cyber physical systems (CPS) [1,2,3,4]. In the process of distribution network intelligence, a large number of intelligent electronic devices (IED) and complex communication links are used in large numbers in the distribution network and play a central role in grid scheduling control and power production management. As the network of these facilities is open, a few unscrupulous individuals make use of their basic knowledge of electrical power systems and the means of cyber attack to launch cyber vulnerabilities against them, thereby profiting from them. If the vulnerabilities of the cyber system are attacked, it will threaten the safe and stable operation of the power systems and cause serious consequences [5]. For example, the 2015 Ukraine blackout, a typical case of cyber attack vulnerabilities, demonstrates that the impact of cyber attack vulnerabilities would be severe; meanwhile, the wide range of strikes showed that the attacker had a precise control of the power system. It has been shown that more precise attacks can be implemented when the attacker possesses certain knowledge of the power system [6]. Currently, information security issues regarding distribution network CPS have received attention from the academic community.

Passive defense is the traditional way to cope with cyber attack vulnerabilities, such as firewall interception and intrusion detection, but the role played by these defense resources is usually limited. To accurately eliminate the impact of cyber attacks, a large number of scientific organizations and scholars have conducted research on CPS cybersecurity issues [7]. Various security risk assessment methods are used to measure and prevent intrusions, typically these security risk assessment methods refer to identifying vulnerabilities, threats and assets in an information facility, accurately and efficiently assessing system risk following an attack, and implementing protection strategies to reduce the negative impact of the threat [8,9]. The literature [10] assessed the most possible scenarios of cyber attack in distribution network CPS using a Bayesian attack graph approach, measuring the risk by the size of consumers disrupted and the duration of the disruption, but did not take into account the attack selectivity of the target node from the attacker’s perspective. Literature [11] analyzes the indirect impact of cyber system failures on the distribution network physical system, which focuses on the fault recovery process of the distribution network CPS and therefore does not consider the impact of cyber attacks on cyber system failures. The literature [12] proposed a multi-model framework for event prediction and risk assessment based on Bayesian network (BN), fault trees, and event trees, but did not integrate the use of the three methods. The literature [13] proposed a quantitative assessment framework that combines the inference process of BN with traditional probabilistic risk analysis and applied it to probabilistic risk assessment of nuclear waste disposal. The literature [14] has developed a power supply restoration model to calculate the consequences of a fake data injection attack on a distribution network CPS, taking into account the interdependence of the cyber and physical system, but it fails to consider the role of the system’s defense resources.

Risk analysis involves inherent uncertainties that incorporate aspects such as the complexity of the system, parameters randomness, the risk model applied, and human subjectivity [15]. The focus of this paper is on proposing a risk assessment method for distribution network CPS that takes into account the attacker’s perspective and the allocation of defense resources, therefore, this paper ignores the uncertainty and propagation problems that exist when modelling Bayesian network. To evaluate the cyber risk problem in distribution network CPS, a dynamic Bayesian network considering the attacker’s perspective is proposed in this paper to quantify the risk of distribution network CPS transmitted from the control layer to the physical layer. Using a one-to-one model of information nodes and physical nodes, Bayesian network is used to straightforwardly portray the risk transfer process from the cyber system to the physical system, taking into account the attacker’s perspective, the attack selection based on the attacker’s knowledge level of the power system, and the defense resources of the system itself. Finally, the risk value of the distribution network CPS is calculated.

In summary, the main works of this paper are as follows:

- Based on the information transfer structure model of the distribution network CPS, the probability of exploiting the vulnerabilities existing in the cyber layer of the distribution network is calculated by using the common vulnerability scoring system (CVSS), and then the Bayesian network model under cyber attack vulnerabilities can be derived.
- Different evaluation metrics are given in this paper to consider attack selection from the attacker’s perspective, not only considering the objective existence of indicator weights, but also incorporating the subjective opinions of several experts who undertake different professional works. Finally, a combination of subjective and objective approaches is used to determine the selection tendency of the attacker, and the practicality of expert experience and the informational variability of objective data are taken into account.
- Multiple scenarios where vulnerabilities in a distribution network cyber system are exploited by attackers are designed and simulated in a dynamic Bayesian network. The dynamic Bayesian network simulation is able to reflect the risk value after an attack vulnerability or under normal conditions, which can reflect whether the system is under attack vulnerability and thus effectively avoid the risk.

## 2. Background of the Study

### 2.1. Structure of Distribution Network CPS

The distribution network CPS is a large integrated system whose physical layer devices and components are supervised and maintained by the network layer transmission control [16].

**Figure 1:**
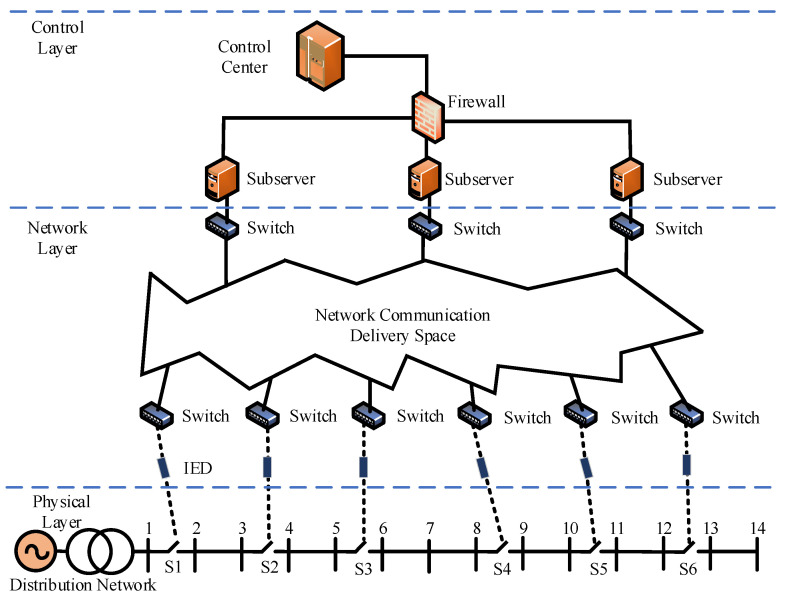
Distribution network CPS model.

According to the different functions of the system devices, a typical distribution network CPS architecture can be divided into three layers as shown in Figure 1 [17]:

- The control layer is an important part of the CPS, whose function is to unify and integrate the data transmitted from different communication networks and generate control commands in response, which guarantees the safe and stable operation of the power system;
- The control layer and the physical layer are connected through the network layer which is responsible for information data transmission during system operation.
- The physical layer mainly consists of power devices and corresponding network components, such as distributed generation units and their controllers, loads and their measurement units, circuit breakers and their devices, and substations and their communication systems.

### 2.2. Cyber Security of Distribution Network CPS

The current cyber layer of the distribution networks are mainly based on an open and networked architecture. Therefore, with the increasing level of intelligence in distribution networks, private networks in distribution systems are increasingly vulnerable to IP-based intrusion attacks, and the security challenges they face are increasing in both physical and cyberspace [18].

The National Institute of Standards and Technology (NIST) reported that the three main factors of network security are confidentiality, integrity, and availability [19], which are often referred to as CIA security objectives, and that cyber attack vulnerabilities on the distribution network cyber layer are achieved through the unauthorized use of network infrastructure in vulnerabilities and security flaws [20], thus disrupting the three CIA elements to achieve a cyber intrusion.

For this paper, the delivery of cyber attack vulnerabilities starts in the form of information downlinked by the compromised vulnerability of the master server. The objective of the attacker is to take control of the physical layer target facility by controlling different levels of vulnerabilities during the attack.

In order to avoid or mitigate the impact of network attacks on the distribution network, the role of security risk assessment is to update the risk value of the system in real time to cut off or defend against network attacks as soon as there is a risk, thereby avoiding the attacker’s strike on the target node and eliminating or minimizing the losses, while, to achieve a more intuitive quantitative risk assessment in this paper, it is possible to avoid to a greater extent the adverse effects caused by network attacks. Security risk assessment is an indispensable part of the safe and stable operation of modern distribution network CPS in summary.

### 2.3. Risk Assessment Process for Distribution Network CPS

**Figure 2:**
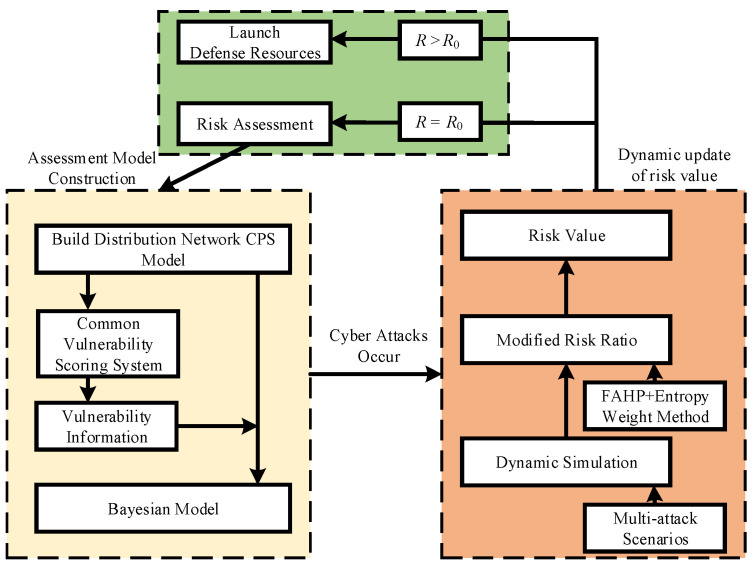
Assessment process.

The process of distribution network CPS risk assessment is shown in Figure 2 below. First, this paper uses CVSS to determine the vulnerability information of the cyber layer of the distribution network according to the architecture of the distribution network CPS to construct a Bayesian network for the information transmission of the distribution network CPS. When the network attack occurs, the corresponding Bayesian dynamic model is constructed to derive the risk probability of the target node under the network attack and, considering the selectivity of the attacker’s perspective on the attacking node, the risk probability of the target node is corrected using subjective and objective assignment to correct the risk probability of the target node. Then, considering the defense resources of the system, the final corrected risk probability is obtained. Finally, the obtained probability is multiplied by the load losses of the node to obtain the quantified dynamic risk value R. For a certain distribution network CPS, the risk value when its vulnerabilities are not exploited by an attacker is called the static risk value $R_{0}$. The magnitude of $R_{0}$ represents the number of vulnerabilities and how easy these vulnerabilities are to be mastered in the distribution network CPS. The dynamic risk value R $\left(R>R_{0}\right)$ indicates that some vulnerabilities of the system have been exploited by an attacker, and the magnitude of this value represents the maximum possible impact of these cyber attack vulnerabilities on the system. A round of risk assessment is completed and compared with the static risk value $R_{0}$, and if it is judged that $R>R_{0}$, the risk has occurred and emergency measures are taken immediately to cope with the risk, and if there is no risk, the next risk assessment is carried out at a certain interval.

## 3. Risk Delivery Model

A risk assessment model needs to be built in order to quantify the risk of the distribution network CPS. This paper uses a dynamic Bayesian network to quantify the risk from its network to the physical layer. Based on the structure of each component of the two systems, the cyber layer and the physical layer of the distribution network CPS, the Bayesian network is used to portray the transmission process of the network risk from the cyber layer to the physical layer.

### 3.1. Common Vulnerability Scoring Systems

In order to obtain the Bayesian network model, the first step is to obtain the probability that the vulnerability of the distribution network cyber system is exploited by the attacker. We used CVSS 3.0 [21] to obtain information about the vulnerability nodes of different devices in the distribution network CPS cyber system through the US national vulnerability database (NVD) [22]. The information on some of these vulnerabilities in the distribution network CPS is shown in Table 1 below.

After obtaining the vulnerability information, the basic evaluation metric information of the vulnerability can be obtained through NVD, and then we can use CVSS 3.0 to obtain the values of each basic metric as shown in Table 2 below.

### 3.2. Bayesian Network Model of Distribution Network CPS

**Figure 3:**
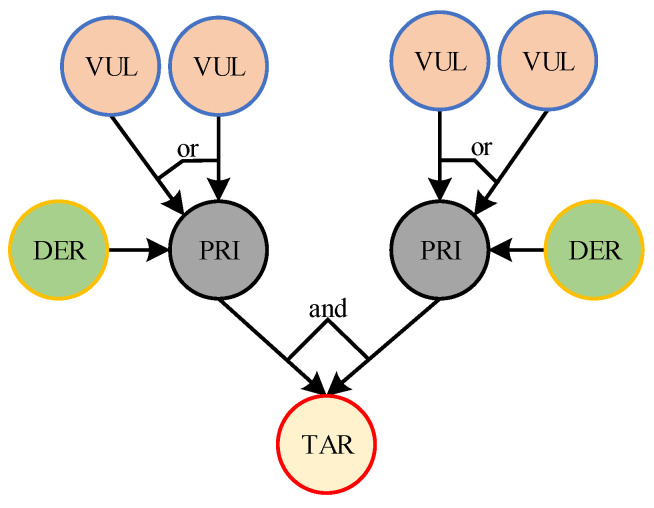
Fundamental Bayesian network of distribution network CPS.

Bayesian network is an uncertainty processing model that simulates causality in the inference process, and its network topology is a directed acyclic graph. In order to use Bayesian networks for security risk assessment of distribution network CPS, the five elements of the Bayesian network model for distribution network CPS are given in Figure 3 as follows [23].

- Attribute Nodes

To reflect the process of distribution network CPS attacks more clearly, the Bayesian network attribute nodes are denoted as $S=\{S_{i}^{N}|i=1,2,\cdots ,n,$N=VUL,PRI,TAR,DER} for risk assessment, where *VUL* denotes the vulnerability node that can be exploited by the attacker in the network system, *PRI* denotes the permission node that the attacker must obtain if he wants to perform the attack operation, *TAR* denotes the target node such as the sensor or actuator that the attacker intends to attack, and *DER* represents the passive defense system. Different attribute nodes indicate different responsibilities or roles generated in particular cyber attack vulnerabilities.

- Directed Edges

The Bayesian network contains directed edges in the directed acyclic structure, where $D=\{d_{ij}|i=1,2,\cdots ,n,j=1,2,\cdots ,n\}$ represents the transfer process from the parent node to the son node.

- Logical Structure

Bayesian networks contain two logical structures: logical ‘and’ and logical ‘or’. Logical ‘and’ means that the son node has the probability to be mastered by the attacker must satisfy that all the parent nodes are captured at the same time, and logical ‘or’ means that the son node has the probability to be mastered as long as either parent node is captured.

- Prior Probability $P_{1}$

Using the basic probability formula, the calculation is obtained based on the logical relationship between the nodes.

- Posterior Probability $P_{2}$

Represents the probability after the dynamic change of each node after a successful attack on the network.

According to the information of the distribution network CPS and the basic Bayesian network model, the five elements of the Bayesian network can be extracted in the process of dispatching information from the main station of the distribution network service. Using a Bayesian network can clearly determine the information transmission process of distribution network CPS in the form of probability transmission, and the interaction process between the cyber system and the physical system; therefore, this paper chooses to use a Bayesian network to assess the risk of distribution network CPS.

In this paper, to show the passive defense function of the distribution network CPS more accurately, we set up a firewall under the main server of the distribution network, whose function is that all vulnerabilities of the main server must be mastered before the main server can be exploited. The occurrence condition of its posterior probability must satisfy that all vulnerabilities of the main server are exploited to weaken the impact of vulnerabilities.

### 3.3. Calculation of Prior Probabilities

#### 3.3.1. Calculate the Probability of Vulnerability Being Exploited

After obtaining the information about vulnerabilities, the formula for calculating the probability of vulnerability being exploited based on the above metrics was derived in conjunction with the literature [24] as follows.

(1) $$P_{e}=2.1\times AV\times AC\times PR\times UI$$

where $P_{e}$ is the probability of the vulnerability being successfully exploited. *AV*, *AC*, *PR*, and *UI* respectively represent the value of the access vector metric, attack complexity metric, privilege required metric, and user interaction metric corresponding to the vulnerability.

#### 3.3.2. Calculation of the Prior Probability

In a non-root node of a Bayesian network, if each of its parents satisfies the ‘and’ relationship, the conditional probability of that node is calculated as follows [25,26].

(2) $$P_{1}(X_{i}|\mathrm{Pa}(X_{i}))=P(\underset{X_{j}^{S}=1}{\cap }e_{i})=\prod_{X_{j}^{S}=1}P(e_{i})$$

where the above equation is 0 if there exists $X_{j}\in \mathrm{Pa}(X_{i})$, $X_{j}^{S}=0$.

In a non-root node of a Bayesian network, if each of its parents satisfies the ‘or’ relationship, the conditional probability of the node is calculated as follows.

(3) $$P_{1}(X_{i}|\mathrm{Pa}(X_{i}))=P(\underset{X_{j}^{S}=1}{\cup }e_{i})=1-\prod_{X_{j}^{S}=1}\left(1-P(e_{i})\right)$$

where the above equation is 0 if for all $X_{j}\in \mathrm{Pa}(X_{i})$, $X_{j}^{}=0$. In the above two equations, $P_{1}(X_{i}|\mathrm{Pa}(X_{i}))$ denotes the conditional probability of node $X_{i}$, $\mathrm{Pa}(X_{i})$ denotes the parent nodes of node $X_{i}$, and $e_{i}$ denotes the event that the vulnerability of node $X_{i}$ has been successfully exploited, and conveys the risk from node $X_{j}$ to $X_{i}$. $X_{j}^{S}$ indicates the status of node $X_{j}$, whether it is successfully leaked or not. $X_{j}^{S}=1$ represents success, and $X_{j}^{S}=0$ represents failure.

### 3.4. Calculation of the Posterior Probability

The above procedure describes only the static conditional probability of the distribution network CPS, and when the attack occurs, its posterior probability is calculated in the following way [26].

(4) $$P_{2}(X_{i})=P(X_{i}|O)=\frac{P(O|X_{i})P_{1}(X_{i})}{P_{1}(O)}$$

where $P(X_{i}|O)$ denotes the probability that node $X_{i}$ is mastered by attackers in the set *O* of security event scenarios; $P(O|X_{i})$ denotes the conditional probability that the security event *O* occurs provided that node $X_{i}$ is in the possession of the attackers; $P_{1}(X_{i})$ denotes the prior probability that node $X_{i}$ is in the grasp; and $P_{1}(O)$ denotes the prior probability that security event *O* occurs.

## 4. Quantitative Risk Assessment of Distribution Network CPS

### 4.1. Portfolio Empowerment Method

In historical cyber attack vulnerabilities on distribution network CPS, a large amount of valid data can be stolen by the attacker and the maximum impact of the attack can be obtained at minimal cost, therefore the selectivity of the strike target should also be taken into account in the risk assessment when the attacker takes control of the zone controller of the distribution network cyber layer. Therefore, assuming that there will be *n* nodes among all attack targets, *m* metrics are used to determine the importance of these nodes. The attack preference correction is performed using a combination of the fuzzy analytic hierarchy process (FAHP) and entropy weight method.

#### 4.1.1. Indicator Definition

The importance of nodes in the network is determined using degree centrality, which is proportional to the importance of the nodes. In this paper, we define the degree centrality a of target node *i* as shown in Equation (5) below.

(5) $$D_{i}=\frac{k_{a}+k_{b}}{N-1}$$

where *N* is the total number of nodes in the grid, while $k_{a}$ and $k_{b}$ are the degrees of the two nodes adjacent to the target node i, which is the number of edges associated with each node. The magnitude of this metric is used to determine the importance of the target node in this paper.

#### 4.1.2. Subjective Weight Based on FAHP

- Calculation Steps

The basic idea of FAHP is to decompose the problem into a hierarchical structure that is composed of a bottom-up multi-level structure based on the characteristics and overall objectives of the multi-objective evaluation problem. Therefore, FAHP decision can be implemented in the following steps [27].

- Build Fuzzy Complementary Judgment Matrix

When comparing two factors in FAHP, the importance of one factor over another is quantitatively expressed, and the fuzzy judgment matrix $A={\left(a_{ij}\right)}_{n\times n}$ is obtained if it has the following properties: $a_{ii}=0.5,i=1,2,\cdots ,n$; $a_{ij}+a_{ji}=1,i,j=1,2,\cdots ,n$. Then, such a judgment matrix is called the fuzzy complementary judgment matrix, and to make the relative importance of any two programs about a criterion quantitatively described, the quantitative scale is usually given by the 0.1–0.9 scaling method as shown in Table 3 below. The ‘On the contrary’ in Table 3 means that if judgment $r_{ij}$ is obtained when element $A_{i}$ and element $A_{j}$ are compared with each other, then judgment $r_{ji}=1-r_{ij}$ is obtained when element $A_{j}$ and element $A_{i}$ are compared with each other.

- Weight Calculation

In practice, the experts of the distribution network will consider the impact degree of each indicator on the target node in the actual operation of the system and use the 0.1 to 0.9 scaling method to judge the importance of these indicators. *k* different fuzzy judgment matrices $A_{1}\sim A_{k}$ are obtained according to the opinions of *k* different experts of the distribution network, where the elements of the fuzzy judgment matrix are $A_{i}={\left(a_{ij}\right)}_{m\times m},i=0,1,\cdots ,k$. The weight of the *i*th metric given by any expert opinion is calculated according to Equation (6) based on the opinions of *k* experts.

(6) $$w_{i}=\frac{\sum_{j=1}^{n}a_{ij}+\frac{n}{2}-1}{n(n-1)}$$

- Consistency Test

A consistency test is performed to verify that the obtained weights are reasonable. Let $w={\left(w_{1},w_{2},\cdots ,w_{m}\right)}^{T}$ be the weight matrix given by the experts and let $w_{ij}=w_{i}/(w_{i}+w_{j})$. Calculate the compatibility index using Equation (7), which is reasonable if it is less than 0.1.

(7) $$I(A,W)=\frac{1}{n^{2}}\sum_{i=1}^{n}\sum_{j=1}^{n}\left|a_{ij}+w_{ji}-1\right|$$

- Subjective Empowerment

The weight matrix $W=(w_{1},w_{2},\cdots ,w_{k})$ is obtained based on the opinions given by the *k* experts, the maximum characteristic root method is adopted to find the average level, and the steps are as follows [28]:

- Set the matrix $F=WW^{T}$;
- Calculate the characteristic root matrix λ and the eigenvector matrix Τ of the matrix F;
- Find the largest characteristic root $\lambda_{\max}$ and its corresponding eigenvector $\theta =(\theta_{1},\theta_{2},\cdots ,\theta_{m})$;
- Normalize the eigenvector ***θ*** to obtain the subjective weight vector $W=(\omega_{1},\omega_{2},\cdots ,\omega_{m})$, $\omega_{j}=\theta_{j}/\sum_{j=1}^{m}\theta_{j}$, given by the *k* experts.

#### 4.1.3. Objective Weight Based on the Entropy Weight Method

The inconsistencies in the magnitudes of the evaluation metrics do not allow for comparison, for which the data are standardized using Equation (8).

(8) $$r_{ij}=\frac{S_{ij}-\min\{S_{ij}\}}{\max\{S_{ij}\}-\min\{S_{ij}\}}$$

where $S_{ij}$ is the value of the *i*th metric at the *j*th node, $\min\{S_{ij}\}$ is the minimum value of the *i*th metric, and $\max\{S_{ij}\}$ is the maximum value of the *i*th metric.

The entropy of the *i*th evaluation metric is shown in Equation (9).

(9) $$e_{i}=-k\sum_{j=1}^{n}f_{ij}\ln f_{ij},i=1,2,\cdots ,m$$

where k=1/lnn, $f_{ij}=r_{ij}/(\sum_{j=1}^{n}r_{ij})$, and assume that $f_{ij}\ln f_{ij}=0$ when $f_{ij}=0$.

The entropy weights are calculated according to Equation (10) as the objective weights of the *i*th metric in the objective weight matrix V.

(10) $$v_{i}=\frac{1-e_{i}}{m-\sum_{j=1}^{m}e_{j}}$$

#### 4.1.4. Combined Weight

After obtaining the subjective weights $W=(\omega_{1},\omega_{2},\cdots ,\omega_{m})$ and objective weights $V=[v_{1},v_{2},\cdots ,v_{m}]$, the combined weights are calculated according to the following Equation (11).

(11) $$z_{i}=\frac{\omega_{i}v_{i}}{\sum_{j=1}^{m}\omega_{j}v_{j}}$$

where $z_{i}$ represents the combined weight of the *i*th indicator, and the magnitude of this value represents the degree of tendency of the *i*th indicator in judging the selectivity of the attacker in attacking the target node.

#### 4.1.5. Attacker’s Selective Probability of Attacking Target Node

In considering the selectivity of the attacker, several different evaluation indicators are selected, and to make the correction value for judging the selectivity of the attacker conform to the objective reality and facilitate the calculation, the corresponding positive ideal interval is set for each evaluation indicator in this paper. The maximum ideal value $M_{i}$ for the *i*th indicator is set, and the minimum ideal value for each indicator is set to 0, so as to obtain the weighted risk rate for correcting attacker selectivity, as shown in Equation (12) below.

(12) $$P_{Zj}=\sum_{i=0}^{m}\frac{S_{ij}}{M_{i}}\cdot z_{i}$$

where $P_{Zj}$ is the strike rate of the *j*th node considering the selectivity of the attacker, $S_{ij}$ is the value of the *i*th metric at the *j*th node, and $z_{i}$ is the combined weight of the *i*th metric.

### 4.2. Risk Quantification Model

To quantify the risk value, this paper replaces the assets size with the target node loads $F_{i}$ at a particular moment in time and calculates the target node $S_{i}$ to quantify the risk value $R_{i}$ according to the following Equation (13).

(13) $$R_{i}=P(S_{i})\cdot F_{i}$$

where $P(S_{i})$ is the risk probability of target node $S_{i}$.

### 4.3. Risk Assessment Flow

**Figure 4:**
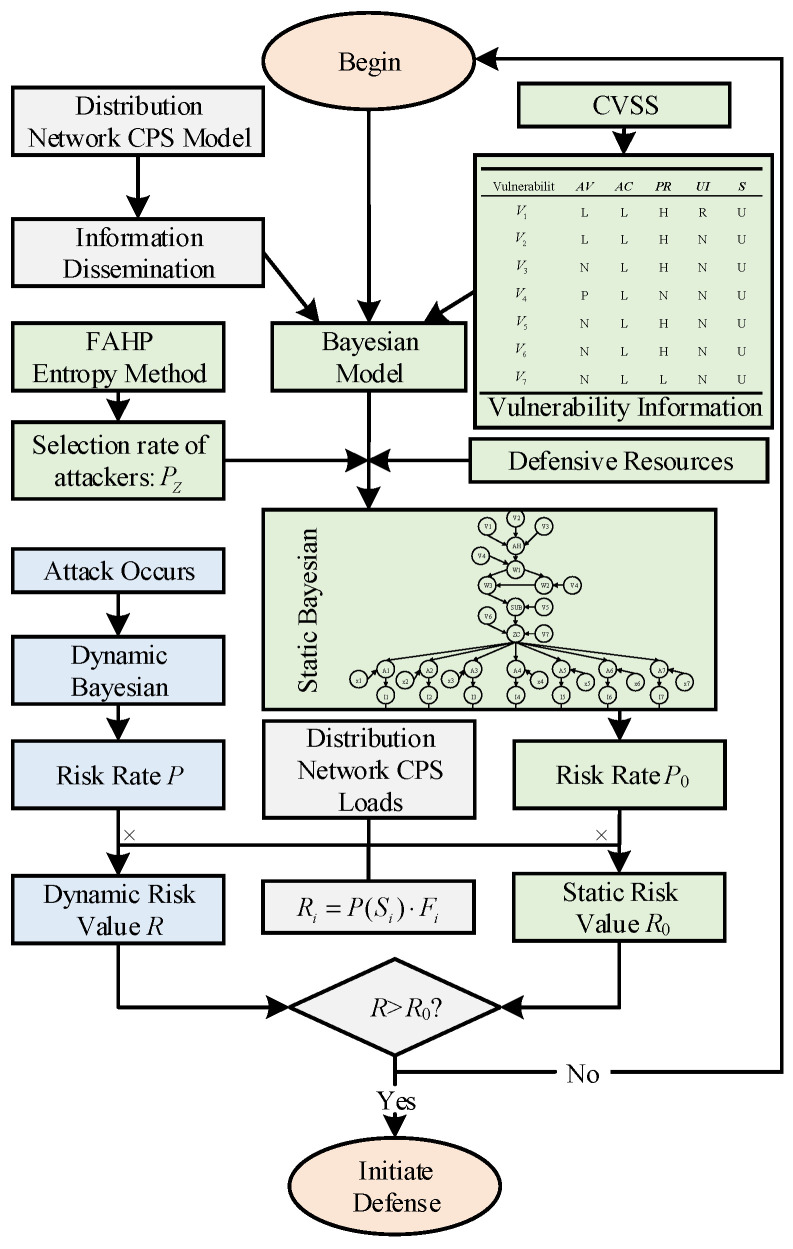
The risk assessment flow of the distribution network CPS.

The flowchart for the arithmetic analysis part of this paper is shown in Figure 4 below based on the above distribution network CPS model, the Bayesian network modeling approach and the risk quantification approach. The dynamic risk value R is compared with the static risk value $R_{0}$ to determine whether the vulnerability is successfully exploited by the attacker in the distribution network CPS after a cyber attack vulnerability occurs.

## 5. Example Analysis

### 5.1. Bayesian Modeling of CPS in Distribution Networks

**Figure 5:**
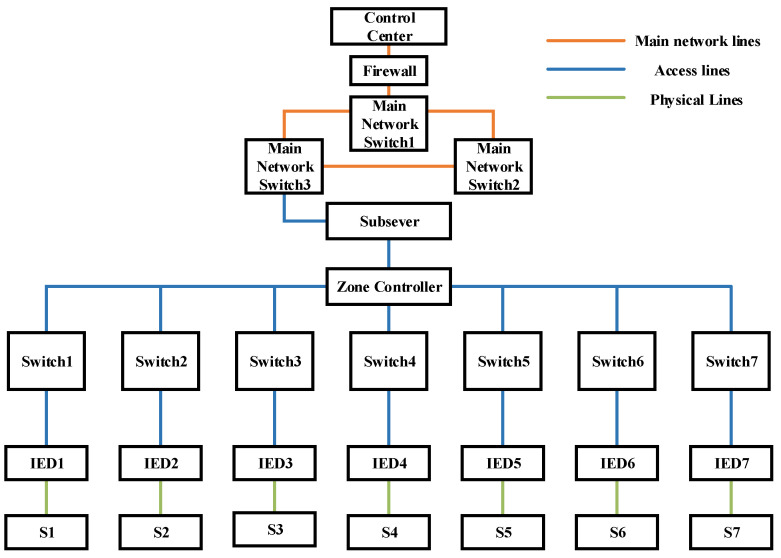
Cyber system of distribution network CPS.

**Figure 6:**
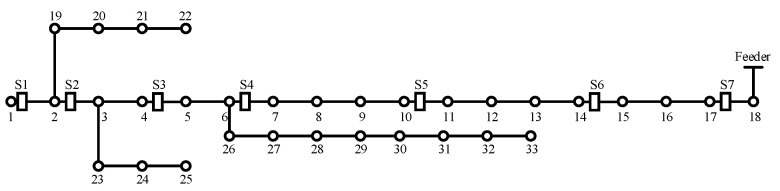
The modified IEEE 33-node distribution network of distribution network CPS.

A distribution network CPS model is established as shown in the following figure referring to the literature [29]. The distribution network CPS is divided into a cyber layer and a physical layer, where the physical system mainly contains traditional primary equipment such as busbars, lines, and switches; the cyber system mainly contains servers, switches, communication lines, distribution-specific security access gateways, and various types of intelligent electronic devices such as remote terminal units, feeder terminal units, and relay protection devices, as shown in Figure 5 below. We use the modified IEEE 33-node system as the physical layer of the distribution network in this paper, and its structure is shown in Figure 6 below. The modified IEEE 33-node system is a benchmark system. In this paper, seven target nodes are set up for the system, and these are presented in the form of switches. The load carried by each target node is the asset model needed to quantify the risk value.

The amount of static load carried by each target node of the IEEE 33-node distribution network system obtained in the simulation is shown in Table 4 below.

To obtain information on the vulnerability of the distribution network CPS, a systematic analysis was carried out. The analysis allows us to find information on some of the most problematic vulnerabilities at the information level. These vulnerabilities are easily exploited by attackers and are not easily fixed by the system. The basic metrics of each vulnerability of the above distribution network CPS cyber system found in the NVD database are shown in Table 5. The exploitable probability P of each vulnerability node is calculated based on Table 1 and Equation (1), as shown in the table below.

**Figure 7:**
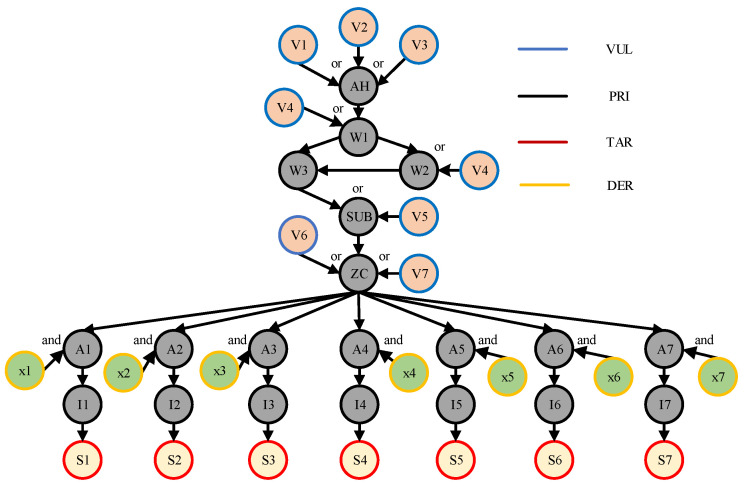
Bayesian network model for distribution network CPS.

The Bayesian network model of the distribution network CPS for the information distribution process is established based on the above distribution network CPS model and the vulnerability information in Table 5, and the defense resources of the system are considered. The Bayesian model and the logical relationship between each node are shown in Figure 7 below.

In the above figure, node AH represents the main server, and the vulnerabilities $V_{1}\sim V_{3}$ are weakened by considering the presence of the firewall in the process of sending its messages. Nodes *W* and *A* represent the switch network, nodes V represent the vulnerabilities that exist at each information station, SUB represents the subsite server, ZC represents the zone controller, nodes *I* represent the IEDs, nodes *S* represent each switch controlled by the IED, which are the target nodes, and its switch corresponds to each node in the physical system of Figure 6, and nodes *x* represent the system self-contained intrusion detection system, human resources, and other defense resources, indicating the processing capability of the system when a network attack occurs. Each node in the network has its own logical structure and each node comes with a conditional probability table, which is calculated by Equations (2)–(4) in different scenarios and different logical relationships.

### 5.2. Simulation Test

#### 5.2.1. Scenario Setting for Different Network Attacks

**Figure 8:**
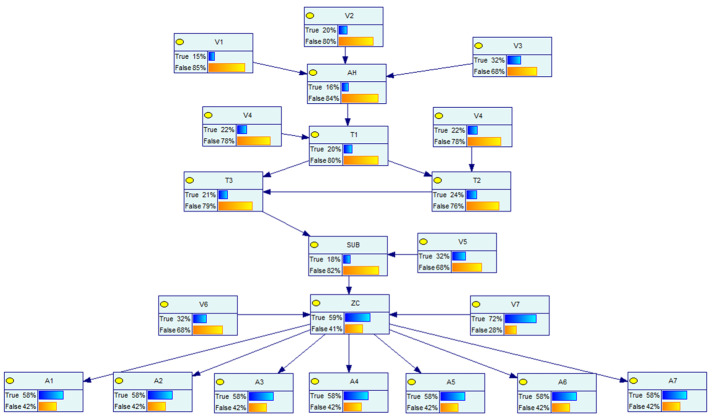
Static Bayesian model.

Based on the above vulnerability probabilities and the Bayesian network model of the information sent down, a static model is built in GeNle as shown in Figure 8. This static scene is set as scene 0.

To verify the dynamic risk of the whole model, three different attack scenarios are set separately.

**Figure 9:**
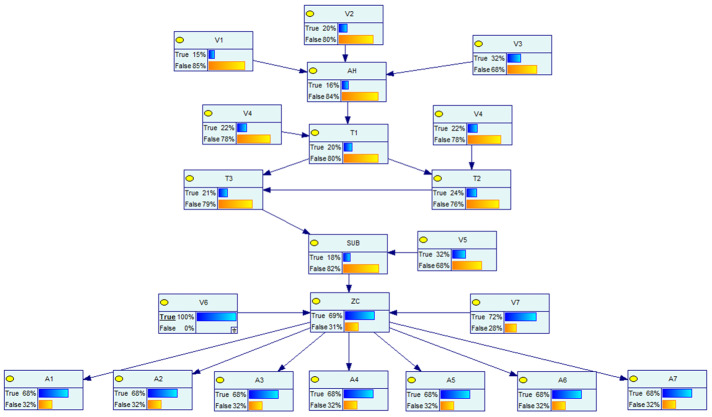
Attack scenario 1.

**Figure 10:**
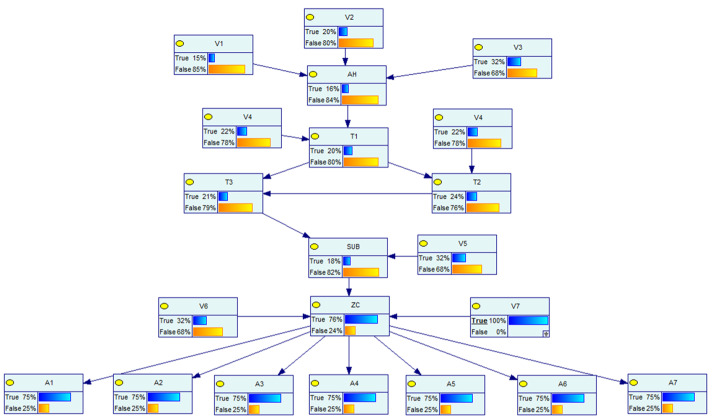
Attack scenario 2.

**Figure 11:**
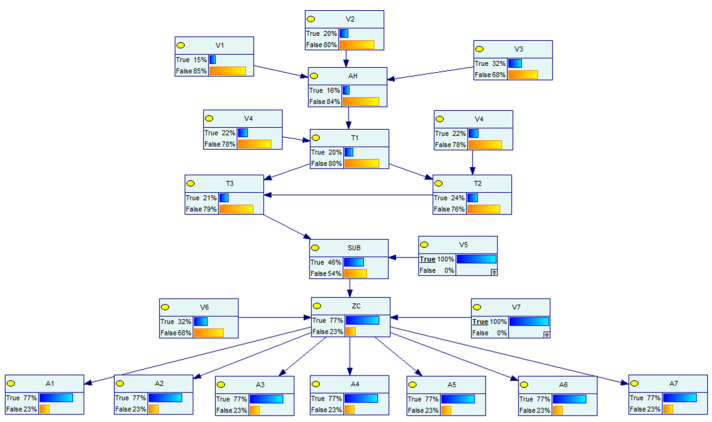
Attack scenario 3.

- The vulnerability $V_{6}$ has been exploited by the attacker, and its dynamic probability is shown in Figure 9, set as scene 1;
- The vulnerability $V_{7}$ has been exploited by the attacker, and its dynamic probability is shown in Figure 10, set as scene 2;
- The vulnerability $V_{5}$, $V_{7}$ has been exploited by the attacker, and its dynamic probability is shown in Figure 11, set as scene 3.

#### 5.2.2. Risk Rate Correction by Defense Resources

When an attack vulnerability occurs, the defense strategy for the probability of the passive defense system to eliminate the impact of risk (risk state 1) is synthesized in this paper based on historical data, expert experience, and the asset value of the system, as shown in Table 6.

#### 5.2.3. Correction of the Selective Strike Target by the Attacker

The three metrics in Table 7 below were selected to assess the attacker’s target selectivity, where metric A is the amount of load carried by the target node, metric B is the number of nodes carried by the target node, and metric C is the degree centrality of the target node, obtained from Equation (5).

- Subjective Weight

For the importance of the above three metrics, six fuzzy judgment matrices were obtained based on the opinions of six experts as shown below.

$$P_{1}=\left[\begin{array}{ccc} 0.5 & 0.6 & 0.7\\ 0.4 & 0.5 & 0.6\\ 0.3 & 0.4 & 0.5 \end{array}\right]P_{2}=\left[\begin{array}{ccc} 0.5 & 0.7 & 0.6\\ 0.3 & 0.5 & 0.7\\ 0.4 & 0.3 & 0.5 \end{array}\right]P_{3}=\left[\begin{array}{ccc} 0.5 & 0.7 & 0.7\\ 0.3 & 0.5 & 0.6\\ 0.3 & 0.4 & 0.5 \end{array}\right]\;P_{4}=\left[\begin{array}{ccc} 0.5 & 0.6 & 0.8\\ 0.4 & 0.5 & 0.6\\ 0.2 & 0.4 & 0.5 \end{array}\right]P_{5}=\left[\begin{array}{ccc} 0.5 & 0.6 & 0.6\\ 0.4 & 0.5 & 0.6\\ 0.4 & 0.4 & 0.5 \end{array}\right]P_{6}=\left[\begin{array}{ccc} 0.5 & 0.6 & 0.7\\ 0.4 & 0.5 & 0.5\\ 0.3 & 0.5 & 0.5 \end{array}\right]$$

The weights of each matrix are calculated using Equation (6) and are shown in Table 8 below.

Using the consistency test to determine whether the expert opinion is reasonable, the results are calculated using Equation (7) as shown in Table 9 below. Here, 0.1 is selected as the test value, once the test indicator *I* is less than 0.1 the opinion is reasonable. All six sets of weights pass the consistency test.

The mean values of the subjective weights were calculated using the maximum characteristic root method as shown in Table 10.

- Objective Weight

The use of Equation (8) to normalize Table 4 is shown in Table 11 below.

The objective weights were calculated using Equations (9) and (10) and obtained as shown in Table 12 below.

- Combined Weight

The combination weights were calculated according to Equation (11) as the importance evaluation weights of the three indicators, which are shown in Table 13.

- Ideal intervals for indicators

To give more reasonable and satisfactory results, ideal intervals are set for three metrics, load: [0, 4544.084], node: [0, 33], and degree centrality: [0, 6/32]. The attack preference probability of the attacker is calculated according to Equation (12), as shown in Table 14 below.

#### 5.2.4. Quantification of Risk

Based on the above study, the risk probabilities of each target node under the static scenario and three attack scenarios are obtained, as shown in Table 15 below.

Finally, the quantitative risk values of each node under different scenarios are calculated according to Equation (13) as shown in Table 16 below.

Due to the existence of vulnerabilities, the probability of risk still exists for the distribution network CPS in normal operation. To accurately assess whether a distribution network CPS is under cyber attack vulnerability, the risk value of each target node in its static scenario must be determined, denoted by $R_{0}^{i}$ as the risk value of the *i*th target node in the static scenario. This value represents the magnitude of the impact of the vulnerabilities on the system. Then, after the *k*th round of risk assessment, if the risk value of node *i* satisfies $R_{k}^{i}>R_{0}^{i}$, the system must have been attacked by the network, and then resources should be mobilized to take corresponding defensive measures to control the risk of node *i*. Meanwhile, it can be seen in Table 15 that the risk values of 7 nodes from left to right increase in order, while in the case of limited resources, the higher the risk value of the node is, the more it should be focused on protection and impose more defensive resources.

Then, we can calculate the risk value of each vulnerability that may be exploited in accordance with this example to derive the degree of impact of different vulnerabilities, with which we can first focus on repairing or regulating vulnerabilities that have a higher degree of impact. For some vulnerabilities that cannot be repaired in a short period of time, it is necessary to increase defense resources to address uncertain cyber attack vulnerability.

#### 5.2.5. Classification of Risk Level

**Figure 12:**
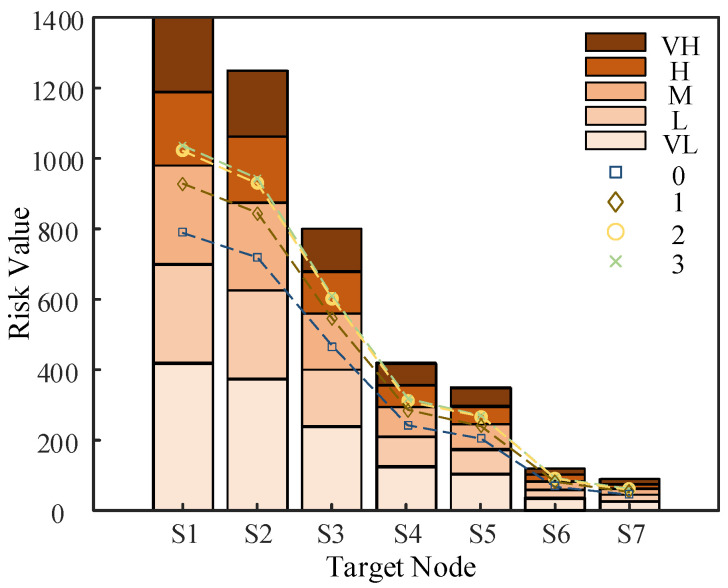
Risk partitioning in different scenarios.

Defensive strategies for dealing with risk should also be differentiated when the size of the risk varies, while there are trade-offs in terms of speed of response and accuracy of elimination. This paper classifies the risk value $R(S_{i})$ of each node in different scenarios of a fully configured distribution network CPS into five different levels: very high (VH), high (H), medium (M), low (L), and very low (VL), and its grading range is shown in Figure 12 below.

Then, the risk partition has been filled in Figure 12 by classifying the risk levels for static scenario 0 and the risk values for the three attack scenarios. The 0 in the figure represents the risk value of each target node under static scenario 0, 1 represents the risk value of each target node under attack scenario 1, 2 represents the risk value of each target node under attack scenario 2, and 3 represents the risk value of each target node under attack scenario 3.

The static risk value of the above distribution network CPS is high according to the above risk partition that can be seen in the medium risk, which requires the adoption of appropriate strategies to optimize or eliminate some vulnerabilities of the system, thereby reducing the impact caused by the system being attacked.

#### 5.2.6. Comparative Analysis of Weight Methods and Defense Resources

To verify the advantages of the combination weighting method and the impact of defense resources, two comparison experiments are made as shown below.

**Figure 13:**
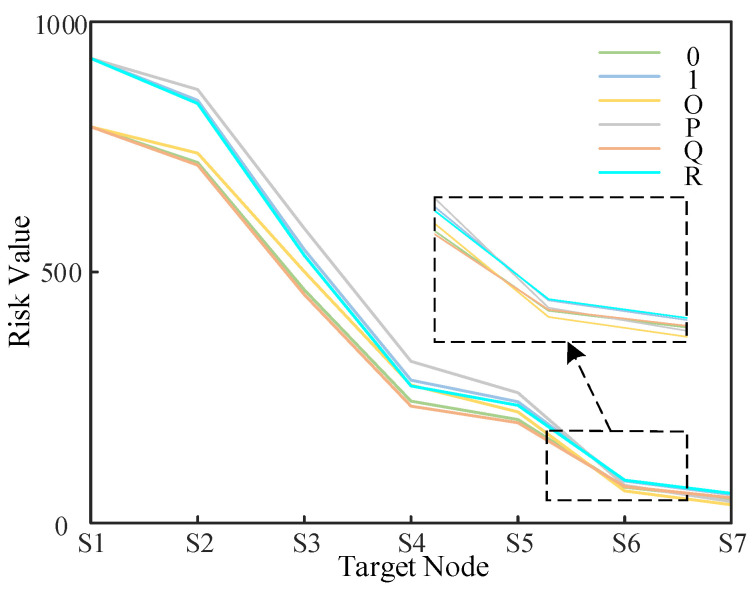
Comparison experiment 1.

**Comparison Experiment 1**: Only FAHP is used in the computational process for static scenario 0 to correct for the attacker’s selection preferences (recorded as scenario O); only FAHP is used in the computational process of attack scenario 1 for the attacker selection preference correction (recorded as scenario P). Only the entropy weight method is used for the selection preference correction in the computational process of static scenario 0 (recorded as scenario Q); only the entropy weight method is used for the selection preference correction in the computational process of attack scenario 1 (recorded as scenario R). The quantitative risk values in the four different scenarios are calculated and compared with static scenario 0 and attack scenario 1, as shown in Figure 13 below.

**Figure 14:**
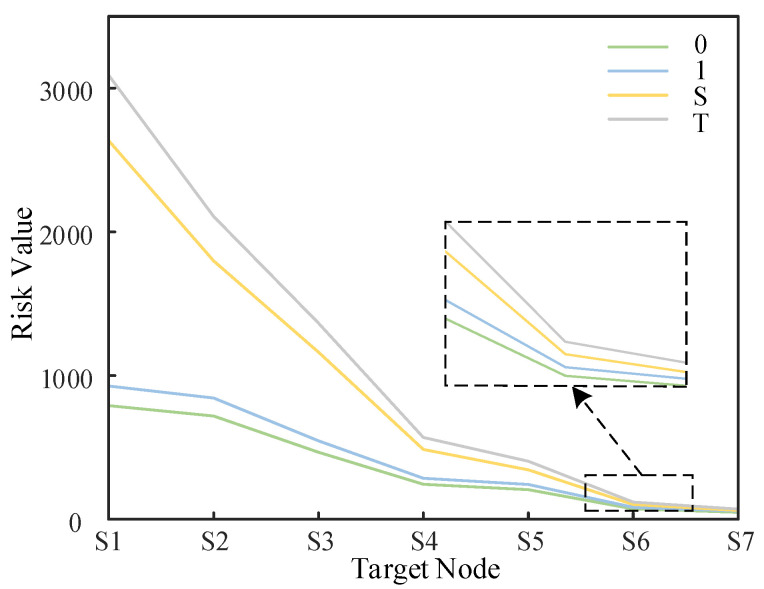
Comparison experiment 2.

**Comparison Experiment 2**: Passive defense is disabled in the computational process of static scenario 0 (noted as scenario S); passive defense fails in the computational process of attack scenario 1 (noted as scenario T). The quantitative risk values in the two different scenarios are calculated and compared with the static scenario 0 and attack scenario 1, as shown in Figure 14 below.

From Comparison Experiment 1, when the choice of subjective opinions of power system experts is considered, the experts generally pay more attention to the risk and will overestimate the actual risk, so that the resulting risk will be higher, which may cause a waste of defense resources. This also shows that subjective weights carry a certain amount of subjective uncertainty. While the value at risk is smaller under objective indicator evaluation, this approach would allow certain potential risks to be ignored, and the subjective selectivity of the attacker judged by the metric value would only consider the objective variability of the metric data at a given moment and would not take into account the uncertainty that may exist in the actual system. The combined use of the two methods overcomes the shortcomings of each, while the weights obtained in this way are more realistic in accordance with the objective variability of the data. The combined use of the two methods will eliminate subjective uncertainty to a certain extent and allow for a more accurate description of the selectivity of the attacker. The rationality of using the combinatorial assignment method to determine the attacker’s selection is verified.

From Comparison Experiment 2, when passive defense resources such as intrusion detection systems failed due to malfunction, the risk value of the distribution network system will be significantly higher after the attack occurred. This further demonstrates the irreplaceable importance of these prevention systems in mitigating risk. Since the role of defense resources is not considered in many risk assessment studies of distribution network CPS, this paper reflects the importance of the defense system through this comparison experiment, so the defense system should be maintained at all times to remain effective. In an open network environment, considering the defense resources of the distribution network system using the method of this paper is an essential part of the security risk assessment, while the allocation of defense resources should also be considered in the case of limited resources.

#### 5.2.7. Base Risk Value Update after Fixing Vulnerability

**Figure 15:**
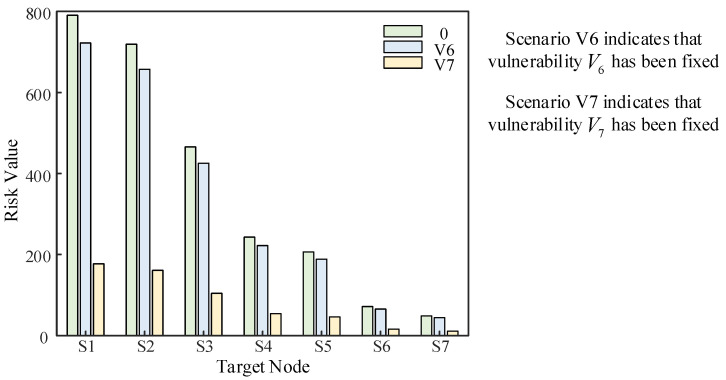
Static risk value after vulnerability fix.

In the above simulation, the value of $R_{0}^{i}$ in static scenario 0 is still large due to the existence of $V_{1}\sim V_{7}$ multiple vulnerabilities. The degree of impact of vulnerability $V_{1}\sim V_{3}$ is weakened due to the presence of firewalls, and the probability of vulnerability $V_{7}$ is the highest according to the above simulation, which has the greatest impact on the assets of the system, so we focus on fixing the vulnerability first. In this section, the vulnerability $V_{6}$ is fixed (recorded as scenario V6), which satisfies $P_{e6}=0$, and the vulnerability $V_{7}$ is fixed (recorded as scenario V7), which satisfies $P_{e7}=0$. A comparison of the risk values of each node in the three static scenarios is shown in Figure 15 below.

From the above figure, the size of the node static risk value represents the size of the ontological risk of a distribution network CPS. The static risk value of the node and the size of the impact on the whole system after being attacked are proportional to the number of vulnerabilities, and the probability of the vulnerability being exploited. Conversely, the smaller the static risk value is, the more stable the system is and the less impact it will have after being attacked. Similarly, after the vulnerability is fixed, the evaluation model should change accordingly and the risk level classification, which is shown in Figure 12 above, should also change accordingly.

**Figure 16:**
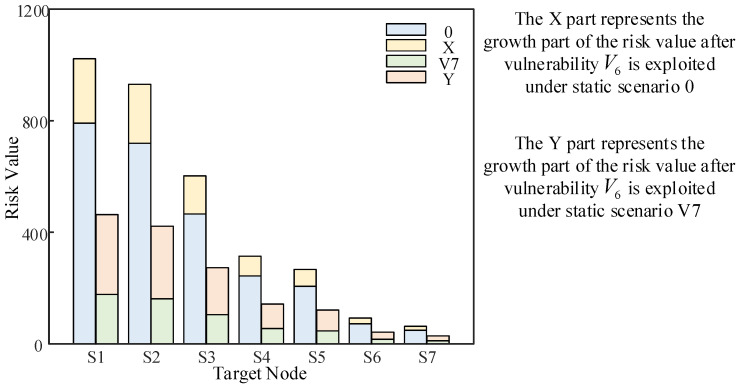
Comparison of the growth in value at risk.

In further analysis, we want to verify how the quantitative risk value of each target node of the distribution network CPS changes when the cyber attack vulnerability is fixed. Figure 16 shows the change in risk value when vulnerability $V_{7}$ is fixed and vulnerability $V_{6}$ is exploited. It is compared with the change in risk value when vulnerability $V_{6}$ is exploited under static scenario 0, as shown in Figure 16 below.

The X part of the above figure represents the growth part of the risk value after vulnerability $V_{6}$ is exploited under static scenario 0. The Y part represents the growth part of the risk value after vulnerability $V_{6}$ is exploited under static scenario V7. The comparison shows that the X part is smaller than the Y part, which indicates that the system with a low static risk value is more affected by the same attack. Therefore, it is important to be vigilant to prevent any vulnerabilities from being exploited by attackers, regardless of whether the static risk value of the distribution network CPS is large or small.

#### 5.2.8. Selectivity of Target Nodes without Considering the Attacker’s Perspective

**Figure 17:**
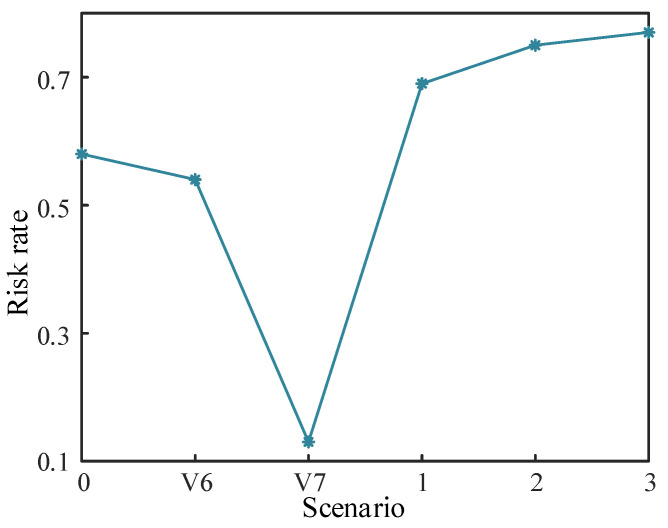
Risk rate of ZC node in different scenarios.

There is uncertainty in the acquisition of weights, and therefore, the selectivity of the attacker for the target nodes can be disregarded. The probability of an attacker exploiting the zone controller (ZC) node at this point is the same as the risk rate passed to each target node. As shown in Figure 17 below, the horizontal axis indicates the probability of the ZC node being controlled by the attacker for static scenarios 0, V6, and V7 and dynamic scenarios 1, 2, and 3. The vertical axis indicates the risk rate of the ZC node in different scenarios.

Since the selectivity of the attacker is not considered, the risk rate of each target node in different scenarios is equal to the risk rate of the ZC node. Instead, the risk value of each target node is simply related to the risk rate of the ZC node and the load carried by the target node. While it does appear to eliminate subjective uncertainty, such a quantified value of risk would be far higher than the value of risk given the attacker’s perspective. This approach can only determine whether a system is under attack by whether the risk rate of ZC node is rising and thus cannot determine which specific target node is being attacked. This would result in a waste of defense resources and greatly increase the cost of defense. As the combined assignment method used in this paper itself eliminates subjective uncertainty to a certain extent, consideration of the attacker’s perspective is an essential part of this paper.

## 6. Conclusions

The dynamic Bayesian network approach is used for risk assessment of distribution network CPS in this paper, which dynamically portrays the impact of different vulnerabilities after they are exploited or fixed by setting a variety of different attack scenarios. Various network attack scenarios can be considered using dynamic Bayesian networks compared to traditional static Bayesian networks. Multiple scenarios are simulated and verified so that the impact caused by different scenarios can be assessed in advance. In addition, corresponding defense strategies are given to quickly and effectively respond to the possible cyber attack vulnerabilities on the distribution network CPS. Based on the case analyses, the following conclusions are drawn.

- Dynamic Bayesian networks that portray cyber attack vulnerabilities in the form of probabilistic transmission are superior in distribution network CPS security risk assessment. It can quantify the risk value of the system when different cyber attack vulnerabilities occur according to different attack scenarios and dynamically calculate the risk value. Based on the size of the risk value, the corresponding defense resources are invested to reduce the impact of cyber attack vulnerabilities.
- From the perspective of the attacker, when it controls the corresponding equipment, there is a certain bias in choosing different strike targets. Using the method of combined assignment to correct this preference can combine the advantages of the subjective assignment method and the objective assignment method to obtain a relatively accurate corrected risk value.
- Risk passive defense resources are an integral part of distribution network CPS. As much as possible, more resources are allocated to nodes with higher risk values in the case of limited resources. The comparison experiments were set up to reflect the role of defense resources in this paper, which demonstrates the need for the defense role to be taken into account in the assessment of security risks in distribution network CPS.
- The magnitude of the static risk value under stable operation of a distribution network CPS depends on the vulnerability information in the system. A system with a high static risk value indicates a greater degree of adverse impact from an attack; however, a system with a low static risk may have a greater increase in risk value from the same attack than the former. Therefore, whenever there is any risk of cyber attack vulnerability in a distribution network CPS, a risk assessment should be carried out.
- As the focus of this paper is to propose a risk assessment method for distribution network CPS that considers the attacker’s perspective and the allocation of defense resources, the uncertainty introduced in Bayesian network modeling and its propagation is not considered. The ways to eliminate uncertainty problems are mentioned in the literature [30]. The physical model in this paper uses a simple distribution network system. In fact, the physical system can be replaced with a more complex distribution network system, such as a distributed generation distribution network, and the method in this paper is equally applicable after replacing the node importance evaluation metric and asset model. Meanwhile, the research can also be extended in subsequent studies to study the optimal allocation of defense resources based on the simulation results, and this series of issues should be considered in further studies.

## Figures and Tables

**Table 1:** Distribution network CPS vulnerability.

Vulnerability Number	Vulnerability Location	CVE Number	Vulnerability Description
$V_{1}$	Control Center	CVE-2021-20106	The vulnerability could allow an administrator user to upload specially crafted files and thus gain administrator privileges on the control center.
$V_{2}$	Control Center	CVE-2021-20135	The vulnerability could allow an authenticated local administrator to run specific executable files on the host.
$V_{3}$	Control Center	CVE-2021-41619	A malicious actor with unmanaged user access on the host could exploit this vulnerability to escalate privileges.
$V_{4}$	Switch	CVE-2022-20864	Enables an unauthenticated, local attacker to recover the configuration or reset the enable password.
$V_{5}$	Sub-server	CVE-2020-12142	Users with knowledge of the system can use the material to decrypt ongoing communications.
$V_{6}$	Zone Controller	CVE-2021-33523	Allows remote upload of a new driver that can execute arbitrary commands on the underlying host.
$V_{7}$	Zone Controller	CVE-2020-5237	Allow remote attackers to upload, copy and modify files on the file system with certain parameters.

**Table 2:** Vulnerability metric information.

Metric	Metric Value	Numerical Value
Attack Vector	N	0.85
	A	0.62
	L	0.55
	P	0.20
Attack Complexity	L	0.77
	H	0.44
Privilege Required	N	0.85
	L	0.62
	H	0.27
User Interaction	N	0.85
	R	0.62

**Table 3:** The 0.1–0.9 scaling method.

Scale	Definition	Description
0.5	Comparing two elements	Equally important
0.6	Comparing two elements	Slightly more important
0.7	Comparing two elements	Obviously important
0.8	Comparing two elements	Much more important
0.9	Comparing two elements	Extremely important
0.1, 0.2, 0.3, 0.4	Inverse comparison of two elements	On the contrary

**Table 4:** Target node load.

Target Node	$S_{1}$	$S_{2}$	$S_{3}$	$S_{4}$	$S_{5}$	$S_{6}$	$S_{7}$
Node Power /MVA	4544.08	3904.53	3346.96	2403.10	1991.01	859.19	632.46

**Table 5:** Probability of vulnerability nodes being exploited.

Vulnerability Number	CVE Number	AV	AC	PR	UI	S	$P_{e}$
$V_{1}$	CVE-2021-20106	L	L	H	R	U	0.1489
$V_{2}$	CVE-2021-20135	L	L	H	N	U	0.2041
$V_{3}$	CVE-2021-41619	N	L	H	N	U	0.3154
$V_{4}$	CVE-2022-20864	P	L	N	N	U	0.2337
$V_{5}$	CVE-2020-12142	N	L	H	N	U	0.3154
$V_{6}$	CVE-2021-33523	N	L	N	N	U	0.9930
$V_{7}$	CVE-2020-5237	N	L	L	N	U	0.7243

**Table 6:** Passive defense probability.

Risk Status	$P(X_{1})$	$P(X_{2})$	$P(X_{3})$	$P(X_{4})$	$P(X_{5})$	$P(X_{6})$	$P(X_{7})$
0	0.7	0.6	0.6	0.5	0.4	0.3	0.2
1	0.3	0.4	0.4	0.5	0.6	0.7	0.8

**Table 7:** The value of each metric.

Meric	$A_{1}$	$A_{2}$	$A_{3}$	$A_{4}$	$A_{5}$	$A_{6}$	$A_{7}$
A	4544.084	3904.529	3346.964	2403.096	1991.011	859.190	632.456
B	33	27	22	12	8	4	0
C	4/32	6/32	2/32	4/32	2/32	2/32	2/32

**Table 8:** Expert weight.

Indicator Node	$W_{1}$	$W_{2}$	$W_{3}$
$a_{1}$	0.3834	0.3333	0.2833
$a_{2}$	0.3834	0.3333	0.2833
$a_{3}$	0.4000	0.3167	0.2833
$a_{4}$	0.4000	0.3333	0.2667
$a_{5}$	0.3667	0.3333	0.3000
$a_{6}$	0.3833	0.3167	0.3000

**Table 9:** Consistency check.

Expert Number	$a_{1}$	$a_{2}$	$a_{3}$	$a_{4}$	$a_{5}$	$a_{6}$
Compatibility Index *I*	0.0554	0.0666	0.0730	0.0664	0.0444	0.0455

**Table 10:** Subjective weight.

Node Metric	$W_{1}$	$W_{2}$	$W_{3}$
Feature Algorithm Value	0.3862	0.3281	0.2857

**Table 11:** Normalized metric.

Indicator	$A_{1}$	$A_{2}$	$A_{3}$	$A_{4}$	$A_{5}$	$A_{6}$	$A_{7}$
A	1	0.8365	0.6940	0.4527	0.3473	0.0580	0
B	1	0.8182	0.6667	0.3636	0.2424	0.1212	0
C	0.5	1	0	0.5	0	0	0

**Table 12:** Objective weight.

Indicator	A	B	C
Entropy Value	0.8234	0.8239	0.5343
Objective Weight	0.2158	0.2151	0.5691

**Table 13:** Combined weight.

Indicator	A	B	C
Objective Weight	0.2158	0.2151	0.5691
Subjective Weight	0.3862	0.3281	0.2857
Combined Weight	0.2633	0.2230	0.5137

**Table 14:** Attack preference probability.

Node	$A_{1}$	$A_{2}$	$A_{3}$	$A_{4}$	$A_{5}$	$A_{6}$	$A_{7}$
A	1.0000	0.8593	0.7366	0.5288	0.4382	0.1891	0.1392
B	1.0000	0.8182	0.6667	0.3636	0.2424	0.1212	0.0000
C	1.0000	0.7500	0.5000	0.2500	0.2500	0.2500	0.2500
Objective Correction	1.0000	0.7883	0.5869	0.3346	0.2890	0.2092	0.1723
Subjective Correction	1.0000	0.8146	0.6461	0.3949	0.3202	0.1842	0.1252
Combined Correction	1.0000	0.7942	0.6000	0.3493	0.2981	0.2049	0.1645

**Table 15:** Risk probability.

Scene	$P(I_{1})$	$P(I_{2})$	$P(I_{3})$	$P(I_{4})$	$P(I_{5})$	$P(I_{6})$	$P(I_{7})$
0	0.1740	0.1842	0.1391	0.1012	0.1036	0.0833	0.0766
1	0.2040	0.2160	0.1631	0.1186	0.1215	0.0976	0.0898
2	0.2250	0.2382	0.1799	0.1308	0.1340	0.1077	0.0991
3	0.2310	0.2446	0.1846	0.1342	0.1375	0.1105	0.1017

**Table 16:** Quantification of risk value.

Scene	$R(F_{1})$	$R(F_{2})$	$R(F_{3})$	$R(F_{4})$	$R(F_{5})$	$R(F_{6})$	$R(F_{7})$
0	790.7	719.2	465.6	243.2	206.3	71.6	48.4
1	927.0	843.4	545.9	285.0	241.9	83.9	56.8
2	1022.4	930.1	602.1	314.3	266.8	92.5	62.7
3	1036.1	942.6	609.8	318.4	270.4	93.7	63.5

## Data Availability

Not applicable.

## References

[1] Liu D., Sheng W., Wang Y., Lu Y. (2015). Key Technologies and Their Progress in Cyber Physics System of Power Grid. Proc. CSEE.

[2] Liu W., Lin Z., Wang L., Wang Z., Wang H., Gong Q. (2020). Analytical Reliability Evaluation of Active Distribution Systems Considering Information Link Failures. IEEE Trans. Power Syst..

[3] Pahwa A., DeLoach S.A., Natarajan B., Das S., Malekpour A.R., Shafiul Alam S.M., Case D.M. (2015). Goal-Based Holonic Multiagent System for Operation of Power Distribution Systems. IEEE Trans. Smart Grid.

[4] Zhuang P., Liang H. (2021). False Data Injection Attacks Against State-of-Charge Estimation of Battery Energy Storage Systems in Smart Distribution Networks. IEEE Trans. Smart Grid.

[5] Liu F., Zhang S., Ma W., Qu J. (2022). Research on Attack Detection of Cyber Physical Systems Based on Improved Support Vector Machine. Mathematics.

[6] Guo Q., Xin S., Sun H. (2016). Integrated Security Assessment of Information Energy Systems from the Ukraine Power Outage. Autom. Electr. Power Syst..

[7] Sridhar S., Hahn A., Govindarasu M. (2012). Cyber–Physical System Security for the Electric Power Grid. Proc. IEEE.

[8] Zhang X., Zhu L., Wang X., Zhang C., Zhu H., Tan Y. (2019). A Packet-Reordering Covert Channel over VoLTE Voice and Video Traffics. J. Netw. Comput. Appl..

[9] Du X., Guizani M., Xiao Y., Chen H.-H. (2009). Transactions Papers a Routing-Driven Elliptic Curve Cryptography Based Key Management Scheme for Heterogeneous Sensor Networks. IEEE Trans. Wirel. Commun..

[10] Dai Q., Shi L., Ni Y. (2019). Risk Assessment for Cyberattack in Active Distribution Systems Considering the Role of Feeder Automation. IEEE Trans. Power Syst..

[11] Zhou X., Yang Z., Ni M., Lin H., Li M., Tang Y. (2020). Analysis of the Impact of Combined Information-Physical-Failure on Distribution Network CPS. IEEE Access.

[12] Zhang Q., Zhou C., Xiong N., Qin Y., Li X., Huang S. (2016). Multimodel-Based Incident Prediction and Risk Assessment in Dynamic Cybersecurity Protection for Industrial Control Systems. IEEE Trans. Syst. Man Cybern. Syst..

[13] Lee C.-J., Lee K.J. (2006). Application of Bayesian Network to the Probabilistic Risk Assessment of Nuclear Waste Disposal. Reliab. Eng. Syst. Saf..

[14] Qin H., Liu D. (2023). Risk Assessment in Distribution Networks Considering Cyber Coupling. Int. J. Electr. Power Energy Syst..

[15] Yazdi M., Zarei E., Adumene S., Abbassi R., Rahnamayiezekavat P. (2022). Chapter Eleven—Uncertainty Modeling in Risk Assessment of Digitalized Process Systems. Methods Assess Manag. Process Saf. Digit. Process Syst..

[16] Song F., Ai Z., Zhang H., You I., Li S. (2021). Smart Collaborative Balancing for Dependable Network Components in Cyber-Physical Systems. IEEE Trans. Ind. Inform..

[17] Cao G., Gu W., Li P., Sheng W., Liu K., Sun L., Cao Z., Pan J. (2020). Operational Risk Evaluation of Active Distribution Networks Considering Cyber Contingencies. IEEE Trans. Ind. Inform..

[18] Wei L., Sarwat A.I., Saad W., Biswas S. (2018). Stochastic Games for Power Grid Protection Against Coordinated Cyber-Physical Attacks. IEEE Trans. Smart Grid.

[19] Pal A., Jolfaei A., Kant K. (2021). A Fast Prekeying-Based Integrity Protection for Smart Grid Communications. IEEE Trans. Ind. Inform..

[20] Zhang Y., Wang L., Xiang Y., Ten C.-W. (2015). Power System Reliability Evaluation With SCADA Cybersecurity Considerations. IEEE Trans. Smart Grid.

[21] Mell P., Scarfone K., Romanosky S. (2006). Common Vulnerability Scoring System. IEEE Secur. Priv..

[22] Johnson P., Lagerström R., Ekstedt M., Franke U. (2018). Can the Common Vulnerability Scoring System Be Trusted? A Bayesian Analysis. IEEE Trans. Dependable Secur. Comput..

[23] Sun Z., Zhang G. (2021). Quantitative Assessment Model for Dynamic Performance Analysis of Security Risks in Industrial Cyber Physical Systems. Control Decis..

[24] Poolsappasit N., Dewri R., Ray I. (2012). Dynamic Security Risk Management Using Bayesian Attack Graphs. IEEE Trans. Dependable Secur. Comput..

[25] Muñoz-González L., Sgandurra D., Barrère M., Lupu E.C. (2019). Exact Inference Techniques for the Analysis of Bayesian Attack Graphs. IEEE Trans. Dependable Secur. Comput..

[26] Laitila P., Virtanen K. (2020). On Theoretical Principle and Practical Applicability of Ranked Nodes Method for Constructing Conditional Probability Tables of Bayesian Networks. IEEE Trans. Syst. Man Cybern. Syst..

[27] Zhang B., Li C.-C., Dong Y., Pedrycz W. (2021). A Comparative Study Between Analytic Hierarchy Process and Its Fuzzy Variants: A Perspective Based on Two Linguistic Models. IEEE Trans. Fuzzy Syst..

[28] Zhang D., Wei K., Yao Y., Yang J., Zheng G., Li Q. (2022). Capture and Prediction of Rainfall-Induced Landslide Warning Signals Using an Attention-Based Temporal Convolutional Neural Network and Entropy Weight Methods. Sensors.

[29] Chen B., Lu Z., Li B. (2019). Distribution Network Reliability Assessment with Multiple Types of Information Disturbances. Autom. Electr. Power Syst..

[30] Yazdi M., Kabir S., Walker M. (2019). Uncertainty Handling in Fault Tree Based Risk Assessment: State of the Art and Future Perspectives. Process. Saf. Environ. Prot..

